# Seeing Through the Façade of Anorexia: A Grounded Theory of Emotional Change Processes Associated With Recovery From Anorexia Nervosa

**DOI:** 10.3389/fpsyt.2022.868586

**Published:** 2022-06-24

**Authors:** Danielle Drinkwater, Sue Holttum, Tony Lavender, Helen Startup, Anna Oldershaw

**Affiliations:** ^1^Salomons Institute for Applied Psychology, Canterbury Christ Church University, Kent, United Kingdom; ^2^Sussex Partnership Foundation Trust, Sussex Education Center, Hove, United Kingdom; ^3^Kent All Age Eating Disorder Service, North East London NHS Foundation Trust (NELFT), Maidstone, United Kingdom

**Keywords:** anorexia, emotion, recovery, emotion regulation, schema therapy, emotion focused therapy

## Abstract

**Objectives:**

Difficulties in managing emotions have been implicated in the development and maintenance of anorexia nervosa (AN), and psychological treatment models seek to address this in putative targets of change. Yet the field of psychotherapy remains unclear and insufficiently evidenced about the process of change and *how* this is actually achieved, including in what steps and in what order within clinical treatment. This qualitative study sought to develop theory about the process of emotional change during recovery from anorexia.

**Methods:**

Semi-structured interviews were carried out with nine women currently engaged in psychological treatment for anorexia. Interviews included questions pertaining to participants’ experience of anorexia, emotions, and emotion management. A constructivist version of grounded theory was employed.

**Results:**

The analysis produced 10 major categories, comprising over 60 focused codes. Categories were clustered together into three super categories, reflecting 3 distinct but interrelated phases of participants’ journeys toward recovery. The phases were: (1) *Coping in a world of uncertainty*, (2) *Seeing through the façade of anorexia*, and (3) *Recovery and growth*. Whilst movement toward later positions often appeared to be contingent on earlier ones, the analysis suggests that this was not an entirely linear process and that participants moved between positions as they grappled with the process of change. Participants came to view behaviors associated with anorexia as emotion-management strategies that were not working and as a façade. As they moved toward recovery and growth, they became less confined by their need for safety, and to see emotions as meaningful and valuable. Becoming more connected to emotional experience and expression, coincided with positive shifts in their intra and interpersonal relationships.

**Conclusion:**

These findings support the recent shift toward emotion-focused models of anorexia. They also highlight an important focus in supporting individuals with AN to connect with, and be guided by, emotional experiences in their relationships with themselves and the world around them. This new grounded theory offers a putative process of change that could be utilized to guide intervention development.

## Introduction

Anorexia is a serious mental health problem, typically developing during adolescence and affecting approximately 0.9% of women over their lifetime (1, 2). People with anorexia engage in dangerous and potentially life-threatening behaviors, including extreme dieting, self-induced vomiting, and excessive exercise, due to extreme weight and body shape concerns (3). Anorexia is associated with poor treatment outcomes and amongst the highest mortality rates of all forms of mental distress (4).

Treatments for AN focus on weight restoration alongside specific psychotherapies. In the UK, the National Institute for Health and Care Excellence (5) recommends specialist psychological interventions be considered for adults with AN, including Eating Disorder Focused Cognitive-Behavioral Therapy (CBT-ED); Maudsley Anorexia Nervosa Treatment for Adults (MANTRA); Specialist Supportive Clinical Management (SSCM); and Focal Psychodynamic Therapy (FPT). Whilst these interventions have shown moderate effect sizes of change (6, 7), it remains the case, even when observed using meta-analyses, that no specialist psychotherapy outperforms any other for adults with AN (8).

Difficulties with emotional processing and emotion regulation are considered transdiagnostic factors within psychological difficulties, including eating disorders (9–14). It is posited that they are significant in both development and maintenance of AN (15–18). Their relevance is emphasized and expanded upon within systematic reviews (12, 14, 19), which highlight a pattern of emotion over-regulation in people with AN, characterized by an overreliance on less helpful emotion regulation strategies, alongside an under reliance upon more helpful strategies, compared to people without a diagnosis (14). Thus, indicating that targeting emotional processing difficulties may be an important treatment focus.

The psychological treatment models for AN use existing psychological theory to address relevant risk or maintaining factors within clinical treatment, affording valuable hypotheses around necessary areas of change. Process-outcome designs test causal theories of which factors predict outcomes and thus can offer support for such theories around targets for change. In AN, such design methods have highlighted treatment targets corroborating the importance of targeting identified emotional difficulties. Potential targets include central symptoms of feeling fat and fear of weight gain (20), motivation to change (21), low self-esteem (22) and social and emotional difficulties, including submissiveness and emotional avoidance/dysregulation (23–25). Qualitative research offers further insights into changes relating to recovery reported by people with AN. It highlights potential treatment targets such as separating the “self” from AN, “reclaiming the self” and achieving self-acceptance or self-reconciliation (26–28).

Whilst it is therefore getting better at highlighting *what* needs to change, the field of psychotherapy remains unclear or insufficiently evidenced about the process of change and *how* that is actually achieved *via* psychological intervention, including in what steps and in what order (29). Indeed, whether specific targeted areas of change are effectively manipulated by interventions as theorized and intended is rarely explored. As such, increasing attention is being paid to the specific components or processes in therapy for eating disorders that better facilitate, or indeed, prohibit change (30). Existing research tends to point to broad therapeutic principles such as feeling understood and maintaining hope (31). Lack of a detailed understanding of the process of change and how this is facilitated is a barrier to developing and improving evidence-based interventions for AN (32) and thus more research is crucial to improving outcomes (33).

Following recent focus on the role of emotions in the development and maintenance of AN (12, 14), and literature regarding identified targets for change described above, the present study sought to explore participants’ subjective experiences of anorexia relating to several key factors linked to therapeutic outcomes, chiefly emotional, and social change in inter and intra-personal relationships. It aimed to develop a theoretical account of the process of change as well as what influences these factors during recovery from anorexia, including any therapy-related change, from the perspective of therapy recipients.

## Materials and Methods

A constructivist version of grounded theory (34) informed the research process, thus an iterative process of data collection and analysis was employed. Comparative methods drew on data to develop conceptual and analytic categories, rather than applying existing theories (34). The use of “constant comparison” (35) enabled data to be revisited and compared and new categories developed. Veering from more traditional forms of grounded theory [e.g., (35, 36)], Charmaz more clearly acknowledges the role of the subjectivity of researchers, and provides specific strategies for addressing researcher biases (see section on “Reliability, Validity, and Reflexivity”).

### Procedure and Recruitment

The study obtained NHS research ethics and R&D approvals (IRAS number- 193479; REC reference - 16/LO/0176 SE Coast Brighton & Sussex). Participants were recruited from two eating disorder services in South East England. Clinicians working in the service were informed about the research and advised of inclusion and exclusion criteria to consider suitability of people on their caseload. Participants were approached by their clinician and given an information sheet, which included sample interview questions. Signed informed consent was obtained.

Theoretical sampling was employed with the aim of developing the properties of emerging categories. Charmaz (34) talks about Theoretical Sampling as helping the research move toward “emergent objectives,” such as to delineate properties of a category, to check hunches, to distinguish or clarify relationships between categories (amongst other things), in order to improve the study, and through increasing precision of categories and making the analysis more abstract and generalizable (p212). This type of approach to research is less concerned with traditional measures of quality associated with the idea of objectivity, and instead looks at other quality standards to demonstrate rigor, such as reflexivity and transparency (37). Because most participants were in the mid stages of treatment at the time of their first interview, information was sought to illuminate categories pertaining to later phases of recovery. Existing participants still engaged with the service who had consented to be contacted for a second interview were invited back and clinicians were contacted again to consider possible participants on their caseloads. Two participants attended a second interview (P6 and P7) and one new participant was recruited (P8). There were approximately 4–6 months between the two phases of interviews.

Inclusion criteria were: (1) outpatients with diagnosis of anorexia nervosa according to DSM-V criteria (3) confirmed by their clinician; (2) Aged = 18; (3) Attending an eating disorder service for therapeutic intervention. Exclusion criteria included: (1) A primary mental health diagnosis other than anorexia; (2) Impaired cognitive function as assessed by a clinician, and (3) high levels of anticipated risk associated with involvement in the research.

### Participants

Twelve participants were approached for the study and agreed to be contacted by telephone (11 during initial phase of interviews and one during the second phase). One participant subsequently declined participation for unknown reasons and two due to reasons relating to their current mental health. Nine participants took part in the study (Table 1), with two participants also attending a second interview. Participants were all females aged between 19 and 61 years. Five of the nine participants were ending their therapy and close to discharge from the service. Therapeutic interventions received by participants included individual or group sessions, largely from a CBT or integrative perspective.

**TABLE 1:** Participant demographic and clinical information.

Participant	Age	Gender	Ethnicity	Diagnoses**	BMI	Marital status	Years since anorexia diagnosis	Length of time with service	Treatment phase
									
P1	20	Female	White British	Anorexia nervosa	15	Single	2 years	1 year	Mid treatment
P2	Missing	Female	Missing	Anorexia nervosa	Missing	Missing	Missing	Missing	Missing
P3	33	Female	White British	Anorexia nervosa	18.5	Single	19 years	3 years	Late phase of treatment
				Substance misuse (H)					
P4	21	Female	Other- mixed	Anorexia nervosa (P)	20	Single/living with partner	4 years	1.5–2 years	Late phase of treatment
				Depression (S)					
				Emotionally unstable personality disorder (S)					
P5	48	Female	White British	Anorexia nervosa (P)	17	Single	31 years	2 years	Late phase of treatment
				Anxiety (S)					
				BPD (S)					
				OCD (S)					
				Depression (S)					
P6	19	Female	White British	Anorexia nervosa (P)	Unsure	Single	4 years	1 year	Late phase of treatment
				Depression (S)					
				OCD (S)					
P7	61	Female	White British	Anorexia nervosa (P)	Unsure	Single	1 year	7 months	Missing
				Post-traumatic stress disorder (S)					
P8	22	Female	White British	Anorexia nervosa	15	Single	3 years	4 months	Mid treatment
								(6 months previously)	
P9	21	Female	White British	Anorexia nervosa	16.5	Single	8 years	2 years	Late phase of treatment

**P, Primary diagnosis; S, Secondary diagnosis; H, Historical diagnosis.

### Interview Schedule

Semi-structured interviews with the lead researcher (DD) were completed face to face and lasted between 45 min and 1 h. The interview schedule was developed by consultation with members of the research team (DD, SH, and AO) and following a full literature review by DD, using key search terms relating to emotion, eating disorders, and emotion regulation.

Warm up questions about participants’ day to day lives afforded the researcher a contextual perspective and provided an opportunity to build rapport (see Supplementary Appendix 1). Participants were then asked questions pertaining to their experience of anorexia, emotions, and emotion management, how these may have changed over time, and what might have influenced such changes. Emerging ideas were followed up and explored. Interviews were audio recorded and transcribed verbatim.

### Data Analysis

The first author transcribed and analyzed all interviewed transcripts following procedures outlined by Charmaz (34) using qualitative analysis software NVIVO. Initial line-by-line coding was employed with the first eight interviews, meaning data content for each line was defined and a code ascribed. This thorough initial coding phase was considered important to refrain from imputing the researchers’ own ideas about relevance. Focussed coding followed; this involved reviewing initial codes and considering their significance, frequency, and relevance. Codes were highlighted, renamed, refined, or collapsed within other codes, tentative categories and subcategories were defined, and links between these categories established. Focussed codes from the initial analysis were applied to data from the three subsequent interviews (two follow-up interviews and one new participant). Existing interview transcripts were also revisited as part of the iterative process of constant comparison (35).

Methodological journal entries and ongoing memo writing throughout the analysis documented the researchers’ decisions, questions, and hunches and guided the analysis of codes and categories and their relationships. Existing theory and literature were applied to the data and incorporated into the memo writing process. Theoretical sufficiency (38) was considered to have been achieved when new data were adequately accounted for by existing categories.

### Reliability, Validity, and Reflexivity

Charmaz (39) acknowledges the subjectivity of the researchers as an intrinsic and inevitable part of the research process. This requires a reflexive stance toward emerging data and the development of theory, including reflecting on one’s values and preconceptions, as well as previous knowledge and experience (39). The lead researcher (DD) had experience working in a variety of mental health settings; however, she considered herself to have little prior knowledge of eating disorders, which also applies to the second and third authors. In line with the constructivist perspective (34); Mays and Pope (37) reflexivity criterion, personal reflections were recorded in the reflective journal and memos.

Quality standards outlined by Mays and Pope (37) were considered throughout. To increase reliability, sections of different anonymized interview transcripts were double coded by two researchers independent of the study (peers who were also trainee clinical psychologists). Differences across interpretations were considered until a consensus was reached. Conversations between the lead researcher and supervisors (second and last authors) about any clinical and theoretical knowledge of the subject matter were deliberately avoided until the final stages of analysis to reduce external influences on data interpretation. A summary of the findings, including a table of codes, categories, and excerpts from participants’ own interviews, was sent out to all participants who had consented, inviting them to provide feedback. Two participants returned feedback which was incorporated into the analysis.

## Results

**FIGURE 1:**
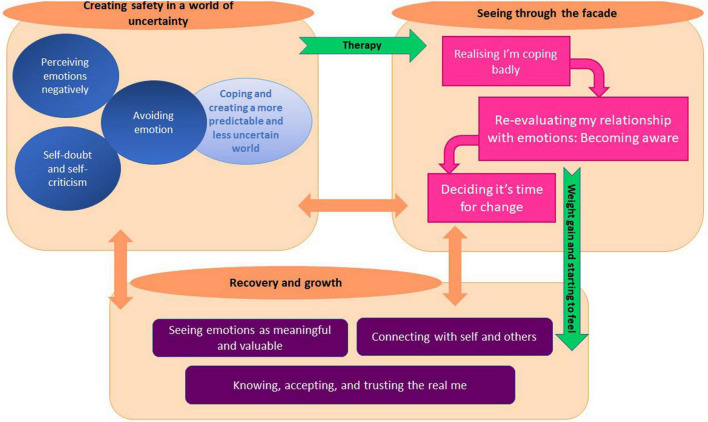
Grounded theory of the change process in recovery from anorexia nervosa.

The analysis produced 10 major categories, comprising over 60 focussed codes. Categories were clustered together into one of three phases of change reflecting a broadly defined phase of participants’ journeys toward recovery: (1) Coping *in a world of uncertainty*, (2) *Seeing through the façade of anorexia*, and (3) *Recovery and growth* (Figure 1). Whilst movement toward later positions often appeared to be contingent on earlier ones, the analysis suggests that this was not an entirely linear process and that participants moved between positions as they grappled with the process of change, particularly between phases (2) and (3). Indeed, at the time of this study participants were in different phases, with some having already moved forward and backward between positions and others showing awareness of what future phases would need to look like, even where not yet experienced. An overview of the grounded theory will be presented before going on to explore each of the categories in more detail (Figure 1).

### Overview of Grounded Theory of Change

Participants spoke of initially developing an emerging insight into their desire to avoid their vulnerability or emotion due to their negative perceptions of emotions, often linked to family narratives, as well as circumstances and life events that led them to feel out of control. Participants became aware that, to avoid emotions and reduce uncertainty, they had developed a host of coping strategies such as detaching, distracting, overcompensating and overcontrolling, which they came to see as not working. This was coupled with high levels of self-criticism and self-doubt which further increased emotion and uncertainty, leading to a greater reliance on problematic coping strategies. Restricting food intake, was seen as one of the many strategies participants relied upon to create a sense of safety, and which fed into and further influenced a vicious cycle of unhelpful coping.

Preceded by a crisis (a time of great distress) or major life event, and finding themselves engaged in therapy, participants were prompted to look at their ways of coping in a new light. An important aspect was gaining an ability to step back to see through the “façade” of the seemingly safe and knowable worlds they had built for themselves. They started to recognize that their strategies were no longer working for them in their lives, and they began to re-evaluate their relationship with their emotions and develop initially external and later intrinsic motivation to change. These new insights coincided with physiological, cognitive, and emotional changes as participants began to gain weight.

Once they had seen through the “façade” of anorexia, participants described various ways in which they began to understand emotions in a new way, as meaningful or valuable. By truly feeling and experiencing emotion and vulnerability, chances for greater connection with themselves and others were facilitated. This appeared to be a key part of the process of getting to know, accept, and trust the “real” self. Categories within *recovery and growth* reflect moments when participants described moving away from their closed off positions so entwined with their “illness,” toward a position of openness, connectedness, meaning, authenticity, and autonomy. They conveyed a sense of being an active participant of life, as opposed to passive recipient.

### Phase 1. Coping in a World of Uncertainty

Participants initially described perceiving their emotions negatively and using different ways of coping to avoid emotion and to create a more predictable and less uncertain world. Many of these ways of coping had become more entrenched once anorexia took hold.

#### Perceiving Emotions Negatively

Participants described how they had begun with negative perceptions of emotions and of thinking that emotions were dangerous, futile, or a weakness; something that interfered with their struggles to survive and to be avoided at all costs. The expression of emotions or vulnerability was viewed as socially unacceptable and perceived by others as melodramatic, selfish or attention seeking. Many related this to family narratives.

*“I think if I was to be a really emotional person then I would get nowhere in life.” [P1]*

*“I think in my family there was a bit of disdain for people who were kind of perceived as being melodramatic or attention seeking.” [P2]*

There was an early recognition that this impacted or impeded an ability to truly be oneself and being very conscious of the impact that might have on other.

*“Growing up I felt I couldn’t have any emotions [*…*] because she [Mum] even said [*…*] P3 if you get down while I’m out and can’t cope it will make me down. So I feel I can’t always be myself.” [P3]*

Even when emotions were felt, participants gave examples of feeling confused, overwhelmed and having little confidence in their autonomy or self-control. They spoke about not understanding their emotions and feeling ill-equipped to manage them.

*“Yeah when I can’t deal with emotions I can’t seem to get my head straight to be able to think about what I’m feeling.” [P4]*

#### Avoiding Emotion

Participants thus spoke of not wanting to experience emotions, and one key way of coping with them was to block or detach from emotions altogether and avoid experiencing them at all. All participants described this. They recounted how not eating further enabled this way of coping, for example as anorexia developed and they became emaciated, they became numb to emotions.

*“When I was like very thin, like emaciated, I didn’t feel anything” [P5]*

Participants also spoke of using certain behaviors to avoid emotions, for example distracting themselves from their emotions (i.e., listening to music) and responding physically (i.e., running off energy). Behaviors associated with anorexia played a significant role, for example, exercise, and dietary restriction as distraction from worries; and purging and laxative use leading to a desired feeling of emptiness. These behaviors provided ways to manage worries and emotions.

*“[Anorexia] reduces your anxiety and worry to [numbers on a scale] instead of like the irreducible complexity of worries about relationships or your place in the world.” [P2]*

#### Coping and Creating a More Predictable World

Participants also described other ways of coping in seeking to create a more predictable and less uncertain world. Participants talked about the importance of routine and structure in their lives, to gain a sense of control and which they associated with reducing uncertainty, particularly following a big change or life event. Anorexia and other planned activities, including shopping, work, and obsessive and compulsive behaviors, gave participants routine and structure, which in turn was linked with a sense of safety and security.

“…*like having to have the structure and doing exercise continuously. I just felt like it kept me safe even though it was incredibly unsafe.” [P6]*

Other aspects of creating more predictability and reducing uncertainty were over-planning, filling time, particularly to avoid thinking and worrying, keeping things simple, and sticking to the rules.

*“Suddenly there wasn’t anyone to give me any rules, so I had to create my own*… *But now I have my rules and I can stick to them”. [P7]*

In many ways, creating the rules enabled participants to develop a greater sense of independence from other people, but also created a sense of detachment from others and stifled the development of a more intrinsic sense of autonomy in which participants would learn to be able to trust their instinct and be more spontaneous. Some felt that it was easier to surrender to others’ judgment or to let them take charge or take control.

*“I mean when I’m in hospital it’s very easy to just do the eating part because someone else is taking the responsibility for it. I’m just doing it.” [P8]*

#### Experiencing Self-Doubt and Self-Criticism

There was an overriding sense that, while participants aimed to reduce uncertainty and emotional vulnerability, it was simultaneously amplified by their regular questioning, self-criticism and self-doubt, such as with regards to the legitimacy of their experience, their judgment, their reasons to feel a certain way and what they felt they deserved to feel or do. The criticism often related to food, weight and shape related themes. This further increased their need and reliance upon the ways of coping they had established.

*“Should I have had that to eat? Should I have not had it? Do I need that to eat? Do I not need it? Have I had too much?” [P3]*

This led to some of the coping described above, such as being subservient or overly dependent upon others. Participants gave examples of feeling confused about their experiences and their behavior, and of having little confidence in their autonomy or self-control.

### Phase 2. Seeing Through the “Façade”

#### Realizing I’m Coping “Badly”

Participants described the next step toward recovery as the realization that, while drawing on this range of strategies to cope, avoid, block, distract from, or displace emotions had felt helpful before, this had limits. Indeed, this phase was about realizing that this pattern was becoming actively unhelpful in their lives and the reasons why. For example, participants recognizing that their coping strategies may help them achieve a quick fix, but that ultimately, they “don’t solve anything” (P3). Participants saw this insight as a positive shift, even if they couldn’t yet make related behavioral change. Participants reflected on the constant swing between ways of coping including using drugs and alcohol (P3; P5), engaging in obsessive routines or behaviors (P6; P7; P2), and harming themselves (P4; P9; P5). P9 likened this to playing the game “whack-a-mole.”

*“I get rid of one bad coping mechanism and another one pops up.” [P9]*

A crucial aspect to this phase was noticing the negative consequences of their coping strategies. This seemed particularly important when participants used multiple dangerous coping strategies, including drug use. They reported experiencing awful consequences from their behavior and recognized that their current strategies were unsustainable. Eventually their coping strategies stopped achieving their purpose and instead made them feel worse. Initial numbness gave way to a build-up of emotions, which ultimately became impossible to hold back.

*“I got stuck in a car park and I just completely started crying. I don’t even really know what happened, but I think I just couldn’t cope anymore” [P6]*

Anorexia often brought a host of other problems and difficult emotions, which participants then had to find ways of managing, increasing their dependence upon problematic coping strategies and their sense of stuckness. For example, taking drugs to stop them thinking about food (P3) or harming themselves in response to feeling guilty for having eaten the “wrong” thing (P4; P9). For P4 and P2, purging was a way of managing feelings of guilt around eating.

Whilst not eating was initially seen as a boost to their ways of coping, participants reported finding that their extreme weight loss led to cognitive and psychological consequences that impacted their mood (P7), their memory and ability to think rationally (P4), their levels of motivation (P9), and their willingness to accept help (P3). Participants came to see anorexia, with its shifting goal posts, as something that was controlling them, as opposed to something which gave them a sense of control.

#### Re-evaluating Relationship With Emotions: Becoming Aware

Participants described how, since they had begun to reflect on their relationship with their emotions, and understand that their current ways of coping were a poor fit in their life, they began to re-evaluate or question their need for these ways of coping in their life as a means to protect them from their vulnerabilities and to consider alternative ways of coping or relating to emotions, often at least in part facilitated by therapy.

*“So yeah part of this process for me has been [*…*] becoming aware of that approach to emotions that I had very much internalised.” [P2]*

Participants described beginning to “notice what other people do” (P9) and “becoming aware that other people did things differently” (P2). For P2, traveling abroad and experiencing other cultures served as a catalyst for later personal change.

“*I met people there who are very [*…*] deliberate and intentional about their feelings and emotions [*…*] I guess that was the moment of becoming aware that um other people did things differently.” [P2]*

Once they had noticed the unhelpful patterns and questioned their role in their life, strong negative perceptions began to loosen. Participants spoke of a shift into understanding that it was okay to have emotions. At the outset, this was experienced as an intellectualized understanding and a shift in cognitive beliefs about emotions.

*“I know in order to get better and to improve my life I have to feel emotions. [It’s] part of life and I’m only just beginning to realise that.” [P5]*

Yet for others, this had progressed further into something more significant, and they spoke of beginning to allow themselves to approach and begin to feel their vulnerability or emotion in small ways.

*“There has been some shift*… *Sometimes yeah I want to feel nothing*… *but I don’t want to feel like that all the time. Whereas I did want to feel like that all the time.” [P4]*

Therapy helped to identify and experience feelings, participants spoke of feeling safer and more in control of their emotions. Putting on weight and starting to experience emotions was still seen as frightening and at times overwhelming, but also considered helpful in facilitating positive change.

#### Deciding It’s Time for Change

When participants spoke about it being time to change, it seemed to emerge in this middle phase of recovery. Participants’ accounts reflected a distinction between external and more intrinsic motivators. The dominant narrative initially, was needing a “legitimate” reason to make positive change and many described the importance of having something or someone else to recover for. P3 wanted to stay well now that she had a daughter, and P7 so that she could care for her dog. Other motivations for changing included physical health, finishing university, and taking advantage of the help that was on offer. Whilst this generally seemed like a positive shift, there was a sense that it was easier, at least initially, to change for others than to change for oneself.

*“It’s harder for people on their own to find the motivation*… *because I don’t have anyone to think about. Apart from my dog*… *He’s kept me alive a couple of times.” [P7]*

These external motivators contrasted with an emerging counter-narrative, reflective of later categories in the recovery process in Phase 3 (below). Later, participants described beginning to recognize their own (emotional) needs as important and deserving of being expressed and met. This led to more intrinsic motivations guided by emotions and associated with themes such as seeking connectedness and meaning. This seemed to have been shaped by the process of seeing through the façade of the anorexia and the ways of coping, and highlighted the shift away from a focus on creating a sense of safety, toward recovery and growth.

### Phase 3. Recovery and Growth

#### Seeing Emotions as Important and Valuable

By phase 3, participants talked of emotions with a sense of meaning related to being human and connecting with others. There was an important shift from intellectualizing about the meaning of emotions, and a realization that emotions should or could be helpful, to now truly appreciating them as meaningful opportunities to open up to the world.

*“I think like the things that generate positive emotions are like also the things that generate negative emotions*… *in the sense of engaging with the world.” [P2]*

Participants were starting to recognize that emotions alert you to important needs; for example, anger helping you to express yourself better (P1), and anxiety alerting you to be more cautious in certain situations (P6). The expression of emotions was connected with feeling better. These shifts in perspective appeared to be contingent upon the learning that took place in therapy, particularly around identifying emotions, and making links between emotions and the unhelpful ways of coping developed to avoid them, including anorexia.

#### Connecting With Self and Others

In contrast to the passive acceptance and ambivalence that was characteristic of “re-evaluating my relationship with emotions” in phase 2, the move toward recovery and growth was tied to participants actively connecting with their emotions. They described going beyond “intellectualizing” (P2) about emotions, toward actually feeling them. Therapy played an important role in facilitating this. P2 spoke about her therapist probing her about how she was feeling and offering empathic conjectures about what emotions she might be experiencing. She also spoke of other strategies she would use to help her connect with how she was feeling, for example journaling. P4 had developed a strategy of talking to her emotions to identify needs associated with the feelings and vulnerability she now recognized.

*“If I’m feeling something so intense and I can’t put my finger on it [*…*] I feel it in my body [*…*] I’ll talk to it and ask it questions like- What are you? Why are you here?”. [P4]*

Participants spoke of the importance of therapy as a safe space to say what is really going on for them, with someone who they felt understood. However, they also began to connect more with others and described having started to talk about how they were feeling to friends and family. Despite having initial reservations about opening-up to others in this way and feeling out of their “comfort zone” (P2), many were surprised to find this helpful.

*“I was a bit*… *like I don’t want to talk about emotions*… *but actually I have ended up talking about my emotions and it has been really helpful.” [P9].*

P8 described how opening up more allowed her to see another way of getting her needs met in more healthy ways than her previous reliance upon unhelpful behaviors to cope and avoiding feeling or showing her emotions.

*“Like actually identify [emotions], actually let them be felt, and share them with other people.”*

Beyond simply talking to other people, participants also expressed an interest more broadly in getting “back into the world” (P5). For P7, there was a link between engaging with those around her and getting to know herself.

*“Once I know who I am that’s going to help in all areas [*…*] in socialising [*…*] with the eating [*…*] coming to terms with who I am.” [P7]*

#### Knowing, Liking, and Trusting “The Real Me”

Through connection with emotion there appeared to be a crucial connection made with the self. P7’s quote above links to this final category in the move toward recovery, highlighting the close relationship between relating to emotions, relating to others and relating to oneself. It seemed important for participants at this phase in their journey to get to know the perceived “real” them and for this to emerge within relationships with others.

*“And the biggest thing for me is I’m beginning to find out a little bit about who I am. The real me.” [P7]*

Participants spoke about learning to like (P3) or accept themselves (P8). These descriptions contrast with how participants spoke of their pasts, for example “not really living as yourself” whilst taking drugs (P3), and keeping up a “façade” of everything being fine (P2). Part of this being the “real me” was about being “okay” with one’s true feelings and to begin to trust in oneself.

*“It’s that confidence to be okay with yourself and your reactions to things.” [P8]*

*“So, once you’ve learnt that the way that you were thinking wasn’t okay*… *you just need to trust yourself.” [P6]*

Participants talked about not having to rely on others (P7; P8), being treated like adults (P9), and growing up (P5). Many referred to therapy as the context in which they were starting to develop a greater sense of autonomy and being able to regulate emotions was seen as an important part of this.

*“I personally think that’s why emotions are SO relevant to my treatment*… *it’ll definitely help me as a person because I’ll be a lot more content*… *able to regulate myself and not rely on other people.” [P8]*

In contrast to the dependency on others and rigid rules described earlier, participants sought flexibility and spontaneity.

*“Recovery is freedom from the structure, from the rules, from the times.” [P7]*

Participants described gaining a new sense of identity. Some spoke of resilience and the importance of feeling good about oneself.

“… *help patients discover what they enjoy/find contentment from*… *a hobby, education, travelling, spending time with others*… *It highlights to someone with little hope that they have something worth living for”. [P9]*

Participants’ accounts reflected that facilitating personal growth and nurturing a more integrated sense of self should be a focus of therapy that may serve as a vehicle for more intrinsic and embedded change (in contrast to intellectualized change).

*“Before a lot of people around me saw me as just P3 with anorexia*… *now they’re seeing me a bit more as something else.” [P3]*

## Discussion

The present study sought to build a theory of the process of therapeutic change, focusing on emotional experience, and how this was facilitated for adults with anorexia during treatment. It broadly found three change process phases that people moved between, and backward and forward within, as they strove to recover from the ED: (1) Coping *in a world of uncertainty*, (2) *Seeing through the façade of anorexia*, and (3) *Recovery and growth.* It highlights a process within which people gain meta-perspective on their previous ways of coping and build a motivation toward finding different ways to manage in their lives. It involves beginning to understand emotions in a new way, as meaningful or valuable and as conduits to enable connection with important relationships and needs previously missed. By truly feeling and experiencing emotion and vulnerability, chances for greater connection with themselves and others were facilitated enabling the emergence of an authentic self that can leave the ED behind. The changed relationship with emotions seemed to be partly prompted by extra-therapy events and circumstances, such as parenthood and in one case exposure to a different and more emotionally expressive culture.

These findings support the premise of the study, namely that change in emotion is of relevance to recovery from anorexia. The experience of participants fits with previous work that reports positive changes in emotional clarity and that goal directed behaviors, even when distressed, relate to improvements in eating disorder psychopathology (40). In addition, these findings align with other studies which highlight a role for “reclaiming” or accepting the self as important in recovery from AN (27, 28, 33). Results corroborate broad themes of empowerment and reconciliation in the process of recovery from anorexia that have been identified elsewhere, where recovery from anorexia was described as a search for identity and truth, and a repossessing of personal control, power and “self” (26). This acknowledgment of “whole self” change necessary for recovery is in keeping with reports that only improving weight is not sufficient for emotion regulation change (41) and that some difficulties with emotion can persist even after resolution of most eating-related difficulties, representing a potential vulnerability for relapse (42). Taken together, we argue this highlights the need for a broader understanding of recovery and therapeutic goals in terms of emotion and ED difficulties. Specifically, the pursuit of an authentic connection with self and identity (“The Real Me”).

The present study adds to previous understanding of emotional change in AN by proposing steps toward achieving this goal. It supports quantitative findings that high levels of emotional avoidance, dysregulation and “submissiveness” in relationships predict poorer outcomes from therapy (23–25). Participants’ experiences link early descriptions of disturbances in perception, inner-doubt and uncertainty, and lack of emotional awareness (43), with the widely reported idea that anorexia, at least initially, helps people to feel in control (44–47). This paper’s account of the process of acceptance and expression of an authentic and emotional self in intra- and interpersonal relationships corroborates suggestions that learning to get alongside rather than avoid anxiety processes, such as emotional uncertainty and self-doubt, is an important step toward reducing reliance on less helpful ways of coping (48, 49).

The findings support previous qualitative research by Fox (50) theorizing early experiences influence later perceptions and management of emotions. They also appear to be in keeping with Schema Therapy (ST) theory. ST proposes that early life experiences, often of chronic unmet need in the realm of relational trauma, may contribute to the development of schemas that become the “lenses through which we view the world” and constrain our behavior accordingly (51). For those with AN, meta-analysis of self-report data indicates that primary core schemas include: Defectiveness, Social Isolation, Subjugation, Dependence/Incompetence, and Emotional Inhibition (14). It is theorized that negotiating the interpersonal world with this belief of “inner defectiveness,” terror of one’s emotional world and profound sense of interpersonal shame, leads to particular coping styles. This may include avoidant coping (i.e., where emotions are squashed *via* starvation and interpersonal retreat), over-compensatory coping (*via* perfectionism and compliance to divert perceived negative judgments from others), and/or resigning to the perceived “defectiveness” (a smaller life with AN). These building blocks mean that there is effectively a “wall of coping” facing the world, rather than an “authentic voice” expressing needs and wishes; this is supported by participant descriptions herein. Thus, there is an ongoing sabotaging of the potential of the “healthy adult” or “core sense of emotional self” to integrate emotional meaning, to get needs met and to flourish (33). The theory of recovery proposed in this paper corroborates this earlier theory of the nature of AN and builds on it by explicating how recovery can happen. It seems important that individuals with AN are supported to gain a “meta-awareness” of their coping patterns that is broader than AN, such that the reliance on coping styles is “loosened,” emotional pain is heard within the therapeutic relationship, and integration of need, emotion and self begins to take place. This supports client perspectives on recovery reported elsewhere which emphasize the desire to be seen and treated as a “whole person” and to build a “real” relationship with a therapist (52).

### Clinical Implications

This paper suggests that clinical change begins with increased “meta awareness” of one’s “parts of self” and core ways of coping. Participants also noted that exploring and understanding how helpful such strategies were was an important basis to be able to subsequently engage in making change. This has clear indications for the focus of therapeutic interventions. Such change processes can be facilitated within existing psychotherapeutic models including CBT and MANTRA. However, gaining meta-awareness and “de-centering” from unhelpful coping patterns (conceptualized as “coping modes”) is a core feature of Schema Therapy (53, 54), indicating that this therapeutic model may be of value in supporting the recovery process outlined herein. The later phases of change described in “Recovery and Growth” reflect cognitive change in one’s beliefs about and relationship with emotion; yet appear to have additional implications beyond cognitive intervention. The “Recovery and Growth” phase describes an internal shift within the person leading toward a connection with emotions in inter- and intra-personal relationships, such that a more authentic self (“the real me”) emerges. This may suggest that therapy should go beyond the traditional, and what could be argued to be surface level, engagement with emotion in therapy, such as targeting cognitive restructuring or building emotional regulation strategies. The factors facilitating emergence of an authentic and trusted self in part related to the opportunity to experientially connect with emotion within therapy. This indicates that therapies offering experiential approaches to emotion, such as Emotion Focused Therapy [EFT; (55)] may be relevant to facilitating such emotional change. EFT seeks to help clients accept, express, regulate, make sense of, and transform emotions, leading to a more resilient and integrated sense of self (56).

### Strengths and Limitations

To the best of our knowledge this is the first study to focus on the process and steps of change in emotions in relation to recovery from anorexia, as opposed to highlighting *what* may need to change. It begins to address important gaps in the theoretical and empirical literature, offering suggestions for clinical practice and further research on how to facilitate change. The small sample size precludes definitive conclusions, and more participants would have allowed for further theoretical elaboration, for example testing the emerging theory with participants belonging to a wider range of cultures and ethnicities, and greater confidence in transferability of findings to such communities. Nevertheless, the interviews were considered to have produced data with enough depth and significance to afford development of the grounded theory, in line with Charmaz (34) guidance, and Dey (38) requirement of theoretical sufficiency. As with other qualitative research, these findings require further exploration and testing.

These findings neither confirm nor disconfirm any one existing therapy/model, but they offer insights into how people with anorexia may come to experience and understand relationships between anorexia and emotions to facilitate recovery. It is possible that certain processes were outside participants’ own awareness and even good quality qualitative research has limitations for developing robust theory to inform effective interventions.

### Further Research

Additional research including participants further ahead in their recovery, and using larger, more heterogeneous samples is needed to elaborate on the differences between individuals’ experiences, and thereby test and extend certain aspects of our hypothesized recovery process. Whilst this study highlighted some aspects of how change was facilitated, further exploration of factors that may influence different processes and trajectories, in particular relating to the perceived contribution of different elements of therapy would be helpful clinically. This could explore broad aspects of therapeutic factors (e.g., empathy, therapeutic alliance), or focus in on specific aspects of the change process and therapist-client interactions in sessions in different phases, to highlight factors such as how best to facilitate the “de-centering” from and gaining meta-perspective on unhelpful ways of coping. The potential impact of participants’ social contexts and any possibilities they may offer for experiencing emotions differently may also be worth exploration. Moreover, triangulating qualitative and quantitative methodologies in future research could help further refine the theory generated here.

## Conclusion

The present study offers a different perspective on emotional and social change in recovery from anorexia, by highlighting not only what needs to change but the process of that change, including a proposed order of change. These three change process phases that people moved between (and backward and forward within) as they strove to recover from the ED indicate aspects of emotional change that may be necessary for recovery and should be considered in future treatment models. It involves beginning to understand emotions in a new way (that is not just intellectualized), as meaningful or valuable, and conduits to a connection with others, and importantly to a connection with the self, such that a knowing and acceptance of self can be achieved and expressed. This highlights useful future directions for development of psychotherapeutic interventions for adults with anorexia.

## Data Availability Statement

The datasets presented in this article are not readily available because this is a qualitative dataset. To protect the identity of participants involved data will not be made available. Requests to access the datasets should be directed to corresponding author.

## Ethics Statement

The studies involving human participants were reviewed and approved by NHS research ethics. The patients/participants provided their written informed consent to participate in this study.

## Author Contributions

DD completed the research project in partial fulfillment of a Doctorate in Clinical Psychology, co-developed the protocol with the lead supervisor AO and obtained ethical approval, conducted the interviews, transcribed and analyzed the data, developed the emerging theory, and contributed to the writing of manuscript drafts. SH was co-supervisor of DD’s thesis, supervised initial qualitative analysis and emergence of categories and theory, read, and contributed to manuscript drafts. TL assisted in developing the concept for the study and supervised qualitative analysis and emergence of categories and theory, and read and contributed to manuscript drafts. HS read and contributed to drafts of the manuscript. AO was supervisor, developed the concept for the study and assisted in study and protocol development and design, consulted on theory and themes once initial analysis was complete and read and contributed to writing of manuscript drafts. All authors read and approved the final manuscript.

## Author Disclaimer

The views expressed in this publication are those of the authors and not necessarily those of the NHS, the National Institute for Health Research, Health Education England or the Department of Health and Social Care.

## Conflict of Interest

The authors declare that the research was conducted in the absence of any commercial or financial relationships that could be construed as a potential conflict of interest.

## Publisher’s Note

All claims expressed in this article are solely those of the authors and do not necessarily represent those of their affiliated organizations, or those of the publisher, the editors and the reviewers. Any product that may be evaluated in this article, or claim that may be made by its manufacturer, is not guaranteed or endorsed by the publisher.
