# Supplementary material for: Seeing Through the Façade of Anorexia: A Grounded Theory of Emotional Change Processes Associated With Recovery From Anorexia Nervosa
Source: Front Psychiatry. 2022 Jun 24;13:868586. doi: 10.3389/fpsyt.2022.868586 (PMC9263079; doi:10.3389/fpsyt.2022.868586)
Supplement: Supplementary file 1 [file Data_Sheet_1.doc]

# Appendix 1- Interview schedule- Grounded Theory

**Situating Q’s-**

- As this is the first time we have met, I wonder if we could begin by you telling me a bit about yourself and your life? *(Descriptive)*
  - Prompts:
    - Living circumstances
    - Relationships
    - Work

**Topic 1- Anorexia**

- As you know, this interview is partly about your experience of anorexia. Can you tell me about the role of anorexia in your life at the moment *(Descriptive)*
  - Prompts:
    - How does it affect you on a day to day basis?
    - How long has it been?
    - How does it impact on your life- living circumstance, relationships, work, health etc.
      - Now?
      - In the past?

**Topic 2- Experiencing emotions / emotion generation**

- Thank you. Now, hopefully you will know from the information sheet about the research that another area I would like to talk about is emotions. Would that be okay? Emotions are something that we all experience and they are part of all of our lives. Some people may think that this is a good thing whilst others may think it is a bad thing. Do you have any thoughts about that? *(Evaluative)*
  - Prompts:
    - How do you feel when you experience intense emotions?
    - What thoughts go through your mind?
    - What does that mean?
    - Can you tell me more about that?
- Do you think other people have the same thoughts or feelings about emotions as you do, or different ones? *(Circular)*
- Has anything changed in the way you think about, or feel about, your emotions? *(Comparative)*
  - Prompts:
    - Have your views changed over time?
    - In what ways?
    - What do you think about that?
    - Is it more helpful or unhelpful to view your emotions in different ways?
    - What has influenced the way you view your emotions?

**Topic 3- Emotion regulation**

- What helps you cope with your emotions?
  - Prompts:
    - (Likely to say something about eating disorder)
    - Can you tell me a bit more about that? How does it help?
    - What else is helpful?
    - Do you ever tell people how you are feeling? Is this helpful/unhelpful?
    - Are there some emotions you feel you can share with others and some you feel you can’t?

Once elicited a few strategies…

- - - Under what circumstances (social) / with you (relational) would you do this?
    - When would you not do this?
    - Are there certain types of emotions, or levels of intensity, where you are more/less likely to do this?

If nothing said about anorexia…

- - - Do you think your anorexia has played a role in helping you to manage your emotions, or not?
    - In what way?
    - Have your views changed on this over time?
    - In what ways?
- Is there anything you've tried in your past that you've found unhelpful to cope with emotions?
  - - Why do you think this was unhelpful?
    - In what ways?
- Have you developed different strategies at different times? (Comparative)
  - Prompts:
    - Why?
    - What do you think might have influenced this change?
      - Experiences/therapy etc.
    - Have you found it is more helpful or unhelpful to manage your emotions in different ways?
- Again, different people have different ways of coping with their emotions. Do you think other people have the same thoughts or feelings managing their emotions as you do, or different ones?
- Do you think that other people use the same strategies to manage their emotions as you do, or different ones? *(Circular)*
  - Prompts:
    - Do you have any thoughts about this?
    - What does that mean to you?

**Debrief**

- So that’s all of the questions that I have for you today. Thank you for taking the time to talk to me. I am aware that we may have covered some sensitive topics. How are you feeling now?
- How have you found speaking with me about your anorexia? And what about speaking about your emotions?
  - Would you like to speak with someone other than me?
- Sometimes people find that they have an emotional reaction to their experience of talking about sensitive topics a little while after the interview. Do you have any support at home or anyone to talk to if this is the case?
- There is additional support you can access through BEAT by visiting [www.b-eat.co.uk](http://www.b-eat.co.uk/) or calling their helpline on 0845 634 1414 between 2-4 pm Monday -Friday.
- Do you have any questions about the research?
- Do you agree to take part in the research? As explained in the information sheet, after today you will not be able to withdraw from the study. However, if you have any concerns please contact me and I will explain further what will happen with your information. The recording will be destroyed once I have transcribed the interview and any identifying information will be removed.
- We would like to send you a summary of the research findings and invite you to give your feedback. Would this be okay?
- My contact details are on the information sheet that I gave to you. Please leave a message for me if you have any questions or concerns and I will get back to you as soon as possible.

Thank you again for your participation.
